# 2024 Acknowledgment of JCM Reviewers

**DOI:** 10.1128/jcm.00367-25

**Published:** 2025-04-09

**Authors:** Alexander J. McAdam

**Affiliations:** 1 Department of Laboratory Medicine, Boston Children’s Hospital, Boston, Massachusetts, USA

## EDITORIAL

The *Journal of Clinical Microbiology*’s high quality and timely publication of groundbreaking scientific research are critically dependent on the outstanding efforts of the editorial board members (https://journals.asm.org/journal/jcm/board-editors) who generously contribute their time, wisdom, and talents to our journal. The editors of JCM also acknowledge the *ad hoc* referees (listed below) who have contributed reviews in the past year, and we express our deep appreciation for their contributions to maintaining the journal’s scientific rigor, relevance of research, and high clinical impact.

Katharina Ackermann

Emily Harris Adhikari

David C. Alexander

Sarah Alexander

Thomas S. Alexander

Roberto Alonso

Melis N. Anahtar

Neil Anderson

Richard M. Anthony

Kotaro Aoki

Derek T. Armstrong

Chris Attaway

Denis Awany

Gad Baneth

Galileu Barbosa Costa

Alan D. Barrett

Allen C. Bateman

Christoph Georg Baums

Karsten Becker

Michael L. Beeton

Robert C. Benirschke

Rebecca Hafkin Berhanu

Mael Bessaud

Sara Blosser

Tim F. Booth

Andrew M. Borman

Valérie Bouchez

David Boxrud

Bobby L. Boyanton

Benjamin Thomas Bradley

John A. Branda

Lucia Maria Almeida Braz

Cameron A. Brown

Ivan Brukner

Kendall Bryant

Noemí Buján

Irene Burckhardt

Susan M. Butler-Wu

Jae-Won Byun

Jianpiao Cai

Melissa J. Caimano

Vitaliano A. Cama

Weiping Cao

Luís Cardoso

Darcie Carpenter

Karen C. Carroll

Dominique A. Caugant

Robin R. Chamberland

Kwok-Hung Chan

Day-Yu Chao

Daniel A. Charlebois

Sudha Chaturvedi

Qu Chen

Bruno B. Chomel

Cornelius J. Clancy

C. Graham Clark

Ryan Close

Joseph M. Colacino

Stephen D. Cole

Courtney Comar

Francois Coutlee

Marc Roger Couturier

Matthew A. Croxen

Karissa Culbreath

Bart J. Currie

Kate Cuschieri

Suzanne E. Dale

Ronit R. Dalmat

David Allan Brett Dance

Olivier Dauwalder

Rodrigo de Almeida Paes

Margaretha de Vos

Ryan Demkowicz

Kanan T. Desai

Alexander Dilthey

Kiril M. Dimitrov

Gerhard Dobler

Haiyan Dong

Michel Drancourt

Steven J. Drews

Cong Duan

Jitender P. Dubey

Edward G. Dudley

P. Dufresne

Robert Duncan

Zoe A. Dyson

Michaela J. Eickhoff

Joseph O. Falkinham

Edward J. Feil

Mark A. Fisher

Thierry Fosse

Ebenezer Foster-Nyarko

Leticia Franco

Dimitrios Frangoulidis

Maria Frediksson-Ahomaa

Inna Friesen

Niels Frimodt-Møller

Heather M. Fritz

Jonathan Gray Frye

Ohad Gal-Mor

Darío Garcia de Viedma

Kenneth Gavina

Ige A. George

Malick M. Gibani

James Gibbons

Chris Gilpin

André Goehler

Richard V. Goering

Wenping Gong

Jose L. Gonzales

Erin Helen Graf

Tristan Richard Grams

Dina N. Greene

Nele Gremmel

Sara B. Griesemer

Thomas E. Grys

Jennifer L. Guthrie

Ferry Hagen

Yohhei Hamada

Mehrad Hamidian

Robert Hamilton-Seth

Axel Hamprecht

Julia D. Hankins

Harley T. Harris

Mohammad Rubayet Hasan

Ronen Hazan

Phillip Heaton

David Hewitt

David Hillyard

Devin B. Holman

Jinlin Hou

Debaki Ranjan Howlader

Daniela Hozbor

Jaroslav Hrabak

Yanyan Hu

Evgeny A. Idelevich

Michael R. Jacobs

Dong-Yan Jin

Bryan S. Kaplan

Sara M. Karaba

Shohei Kawaguchi

Anusak Kerdsin

Sarah E. Kidd

Paul R. Kinchington

Jeffrey D. Klausner

Kalmia E. Kniel

Laura M. Koeth

Irina Kontsevaya

Colleen Suzanne Kraft

Jeffrey Kubiak

Peter Kuhnert

Ed J. Kuijper

Kathy Kurth

Katrien Lagrou

Laura Faye Lalonde

Daryl M. Lamson

Jacob E. Lazarus

Christine C. Lee

Rogan Lee

William T. Lee

Jamie K. Lemon

Bruce R. Levin

Wentao Li

Xunde Li

Rachael M. Liesman

Paul M. Luethy

Oliver Lung

Adrian J.F. Luty

Barry R. Lutz

Angela Ma

Emily MacLean

Burkhard Malorny

Nicasio Mancini

Brendon Coenrad Mann

M. David Mansouri

Jamie Marino

Rebecca Marrero Rolon

Yasufumi Matsumura

Max Maurin

Todd P. McCarty

Bruce A. McClane

David McGivern

Timothy Daniel McHugh

Conor Meehan

Anna E. Merrill

Frank A. Middleton

Anisha Misra

Jose Gilberto Montoya

Mayilsamy Muniaraj

Gina K. Munier-Thomson

Gemma G. Murray

Kimberlee A. Musser

Samia N. Naccache

Subhadra Nandakumar

Jessica Navero Castillejos

Roel H.T. Nijhuis

Matthew Nonnenmann

Patrice Nordmann

Andrew P. Norgan

Anne-cécile Normand

Robert Norton

Randall J. Olsen

Aharon Oren

Volkan Özenci

Elizabeth L. Palavecino

John R. Papp

Bijal A. Parikh

Lindsay A. Parnell

Logan Patterson

Helene M.M. Paxton

Herve Pelloux

Michael Pentella

Jeannine M. Petersen

Dylan R. Pillai

Mario Poljak

Mark Aaron Poritz

Gary W. Procop

Bonnie Chase Prokesch

Nicole E. Putnam

Yvonne Qvarnstrom

Harisha Ramachandraiah

Mitchell D. Ramuta

Jacob Rattin

Florence Robert-Gangneux

D. Ashley Robinson

Eduardo Rodríguez-Román

David Rodriguez-Temporal

Stephen J. Rogerson

Raymond R. Rowland

Jenna Rychert

Orhan Sahin

Halima Said

Max Salfinger

Vittorio Sambri

Linoj Philip Samuel

Maurizio Sanguinetti

Jessica Santos Streauslin

Michael Joseph Satlin

Patrick D. Schloss

Kate Schumann

Inderpaul Singh Sehgal

Inna Sekirov

Arlene Sena-Soberrano

Amir Seyedmousavi

Susan E. Sharp

Hiroyuki Shimizu

Jaya Shrivastava

David Šmajs

Derek D.N. Smith

Emily Snavely

Akos Somoskovi

Sveinung Wergeland Sørbye

Bram Spruijtenburg

Christen Rune Stensvold

Alana K. Sterkel

Yvon Sterkers

Linda M. Styer

David Sue

Kaede V. Sullivan

Wellington Sun

Yi-Wei Tang

Lili Tao

Keith Tardiff

Nicole J. Tarlton

Fred M. Tatum

Deepanker Tewari

Richard (Tom) B. Thomson

Robert J. Tibbetts

Edine W. Tiemersma

Maria Lina Tornesello

Arthur H. Totten

Anthony Tran

Eija Trees

Thao T. Truong

Nicholas A. Turner

Coretta Van Leer Buter

Olivier Vandenberg

Thara K. Venkatappa

Lucio Vera-Cabrera

Debra A. Wadford

Gabriel E. Wagner

Taylor A. Wahlig

Christian Walter

Hannah Wang

Huanyu Wang

Kelly A. Weedmark

Kosala Gayan Weerakoon

Bart C. Weimer

Henrik Westh

Nils Wetzstein

Philip Lewis White

Ryan R. Wick

Rebecca P. Wilkes

Carrie Wilson

Gwendolyn E. Wood

Chi-Jung Wu

Lan Xu

Reina Yamaji

Ming Yang

Shangxin Yang

Eugene Y.H. Yeung

Mitsunori Yoshida

Tetsushi Yoshikawa

Sean X. Zhang

Shuai Zhang

